# Normal product form of two-mode Wigner operator

**DOI:** 10.1038/s41598-022-06124-8

**Published:** 2022-02-14

**Authors:** Rui He, Xiangyuan Liu, Xiangfei Wei, Congbing Wu, Gang Zhang, Min Kong

**Affiliations:** 1 College of Electrical and Photoelectronic Engineering, West Anhui University, Luan, 237012 Anhui China; 2 State Key Laboratory of Pulsed Power Laser Technology, School of Electronic Countermeasures, National University of Defense Technology, Hefei, 230031 Anhui China

**Keywords:** Quantum mechanics, Theoretical physics

## Abstract

In the context of normal product, we use the method of the integration within an ordered product (IWOP) of operators to derive three representations of the two-mode Wigner operator: SU(2) symmetric description, SU(1,1) symmetric description and polar coordinate form. We find that two-mode Wigner operator has multiple potential degrees of freedom. As the physical meaning of the selected integral variable changes, Wigner operator shows different symmetries. In particular, in the case of polar coordinates, we reveal the natural connection between the two-mode Wigner operator and the entangled state representation.

## Introduction

In quantum theory, according to the Heisenberg uncertainty principle, one cannot accurately measure the position and momentum of a particle at the same time, that is, one cannot determine a phase point in the phase space. Therefore, people naturally think of defining the quasi-distribution function in phase space to study the quantum state and motion of microscopic particles. In 1932, Wigner introduced a quasi-classical distribution function *W*(*q*, *p*) corresponding to the density operator $\rho$^1^, the marginal distributions of which corresponds to the particle probability measured in the coordinate *q* space and momentum *p* space, respectively. This gave the phase space a new meaning and opened the front page of phase space quantum mechanics.

In order to calculate the Wigner function of different quantum states, it is necessary to introduce the Wigner operator. Generally, if we know the density matrix $\rho$ of a system’s quantum state, we can calculate the Wigner function *W*(*q*, *p*) of the system by tracing the product of the density operator $\rho$ and the Wigner operator (Wigner kernel) $\Delta (q,p)$

1 $$\begin{aligned} W(q,p)=Tr[\rho \Delta (q,p)]. \end{aligned}$$

That is, for any state, the Wigner function of the system is the expected value of its Wigner operator. For any pure state $\rho =|\psi \rangle \langle \psi |$, the Wigner function of the system is $W(q,p)=\langle \psi |\Delta (q,p)|\psi \rangle .$For any mixed state $\rho =\underset{\psi }{\sum }p_{\psi }|\psi \rangle \langle \psi |$ , the Wigner function of the system is $W(q,p)=\underset{\psi }{\sum } p_{\psi }\langle \psi |\Delta (q,p)|\psi \rangle .$

In classical optics, two orthogonal harmonic oscillators generate an elliptical motion, which is the simplest Lissajous figure. In the phase space of quantum optics, two orthogonal harmonic oscillators can be properly described as the product of the corresponding Wigner operators

2 $$\begin{aligned} \Delta (\alpha ,\beta )=\Delta (\alpha )\Delta (\beta ), \end{aligned}$$

$\Delta (\alpha )$ and $\Delta (\beta )$ are the Wigner operators of single-mode harmonic oscillator in the *x* and *y* directions, respectively. $\Delta (\alpha ,\beta )$ is actually a two mode displaced parity operator, and its general form is

3 $$\begin{aligned} \Delta (\alpha ,\beta )=D(\alpha )(-1)^{a^{\dag }a}D^{\dag }(\alpha )D(\beta )(-1)^{b^{\dag }b}D^{\dag }(\beta ), \end{aligned}$$

where $a^{\dag }$, *a* are creation and annihilation operators respectively, $D(\alpha )$ is the standard displacement operator and $D(\alpha )=\exp (\alpha a^{\dag }-\alpha ^{*}a)$. In Ref.^2^, through observing the correlations described by the Wigner function of the Einstein-Podolsky-Rosen state in the joint measurement of the operator $\Delta (\alpha ,\beta )$, they demonstrated the Wigner function provided direct evidence of the nonlocal character of this state.

In Ref.^3–5^, the normal product form of two-mode Wigner operator was given as

4 $$\begin{aligned} \Delta (\alpha ,\beta )= & {} \frac{1}{\pi ^{2}}:e^{-2(\alpha ^{*}-a^{\dag })(\alpha -a)}e^{-2(\beta ^{*}-b^{\dag })(\beta -b)}:\nonumber \\= & {} \frac{1}{\pi ^{2}}:\exp [-2(a^{\dag }a+b^{\dag }b)+2\alpha a^{\dag }+2\beta b^{\dag }+2\alpha ^{*}a+2\beta ^{*}b \\&-2(|\alpha |^{2}+|\beta |^{2})]:, \end{aligned}$$

where $::$ represents the normal product symbol. The focus of this article is to explore the characteristics of the normal product form of the two-mode Wigner operator. Our main purpose is to integrate over the trivial parameters in the two mode Wigner operator by using the method of the integration within an ordered product (IWOP) of operators^6,7^, and then discuss the integrated Wigner operator and its Wigner function. There are several previous works^8–10^ that have performed integration on the two-mode Wigner operator, but they are not integration within the normal order of the operators and the calculations are more complicated. Generally speaking, people have not noticed the superiority and potential application value of the normally ordered form of two-mode Wigner operator. However, as far as our research is concerned, the normal product form of the Wigner operator has at least two advantages in applications. On the one hand, the operator can be regarded as a *c*-number in the normal product. Therefore, one can freely integrate without considering the noncommutability of the operator, which greatly simplifies the complexity of the problem. On the other hand, by using the property of normal product $\langle z|:f(a,a^{\dag }):|z^{\prime }\rangle =f(z^{*},z^{\prime })\langle z|z^{\prime }\rangle$, the expected value of Wigner operator in the coherent state, i.e., its Wigner function, can be easily obtained. Furthermore, in principle, by inserting the completeness of the coherent state, the Wigner function in any pure state can be calculated.

Our work is arranged as follows: In “Two-mode Wigner operator for SU(2)” section we start from the viewpoint of the polarization of light. We consider the superposition of two mode light in two orthogonal directions, say *x* and *y*, and introduce the specific parametrization of $\alpha$ and $\beta$ with certain physical meanings. After getting rid of the variables not related to the polarization, we derive the normal product form of the Wigner operator. Through some subtle transformations, we obtain the SU(2) symmetry description of Wigner operator, which is the same results in Refs.^8,9^. Then, we immediately give its Wigner function at the coherent state. In “Two-mode Wigner operator for SU(1,1)” section, firstly, we re-parameterize $\alpha$ and $\beta$ with different physical meanings. Then, similar to “Two-mode Wigner operator for SU(2)” section, we obtain the normally ordered form of the Wigner operator in this case by integrating over the unphysical parameters. Next, by applying the properties of Weyl ordering under similar transformation, we get the SU(1,1) symmetry description of Wigner operator, which is consistent with the result in the Ref.^10^. In “Two-mode Wigner operator in polar coordinates” section, we parameterize $\alpha$ and $\beta$ in the case of polar coordinates and integrate over the radius and angle variables respectively. Then, we obtain the marginal distribution of the two-mode Wigner operator, which proves that the result is exactly the pure state density matrix of the entangled state representation.

## Two-mode Wigner operator for SU(2)

In classical optics, a Lissajous figure needs only three independent quantities to be fully characterized: the amplitudes of each oscillator and relative phase between them. Without loss of generality, we introduce the parametrization

5 $$\begin{aligned} \alpha =re^{i\chi }\cos \frac{\theta }{2},\beta =re^{i\chi }e^{-i\varphi }\sin \frac{\theta }{2}, \end{aligned}$$

where $\chi$ is a global phase, the radial variable

6 $$\begin{aligned} r^{2}=|\alpha |^{2}+|\beta |^{2} \end{aligned}$$

represents the total intensity, and the parameters $\theta$ and $\varphi$ can be interpreted as the polar and azimuthal angles, respectively, on the Poincare sphere: $\theta$ describes the relative amount of intensity carried by each mode and $\varphi$ is the relative phase between them^9^.

Substituting Eq. (5) into Eq. (4), $\Delta (\alpha ,\beta )$ can be recast as

7 $$\begin{aligned}{}&\Delta (r,\chi ,\theta ,\varphi ) \\ \equiv & {} \Delta (\alpha ,\beta ) \\= & {} \frac{1}{\pi ^{2}}e^{-2r^{2}}:\exp [2re^{i\chi }(\cos \frac{\theta }{2}a^{\dag }+e^{-i\varphi }\sin \frac{\theta }{2}b^{\dag }) \\&+2re^{-i\chi }(\cos \frac{\theta }{2}a+e^{i\varphi }\sin \frac{\theta }{2} b)]\exp [-2(a^{\dag }a+b^{\dag }b)]:\nonumber \\= & {} \frac{1}{\pi ^{2}}e^{-2r^{2}}:\overset{\infty }{\underset{k=0}{ \sum }}\frac{[2re^{i\chi }(\cos \frac{\theta }{2}a^{\dag }+e^{-i\varphi }\sin \frac{\theta }{2}b^{\dag })]^{k}}{k!} \\&\times \overset{\infty }{\underset{k^{\prime }=0}{\sum }}\frac{ [2re^{-i\chi }(\cos \frac{\theta }{2}a+e^{i\varphi }\sin \frac{\theta }{2} b)]^{k^{\prime }}}{k^{\prime }!}\exp [-2(a^{\dag }a+b^{\dag }b)]: \\= & {} \frac{1}{\pi ^{2}}e^{-2r^{2}}:\overset{\infty }{\underset{ k,k^{\prime }=0}{\sum }}\frac{[2r(\cos \frac{\theta }{2}a^{\dag }+e^{-i\varphi }\sin \frac{\theta }{2}b^{\dag })]^{k}[2r(\cos \frac{\theta }{ 2}a+e^{i\varphi }\sin \frac{\theta }{2}b)]^{k^{\prime }}}{k!k^{\prime }!} \\&\times e^{i\chi (k-k^{\prime })}\exp [-2(a^{\dag }a+b^{\dag }b)]:. \end{aligned}$$

After writing $\Delta (r,\chi ,\theta ,\varphi )$ as the normal product form of Eq. (7), we can use the IWOP method ^6,7^ to perform the integral operation of the operator. Since all Bose operators are in  :   :  internally, they can be treated as integration parameters, so that the integration can proceed smoothly. In order to eliminate the variables unrelated to the idea of polarization (i.e., total intensity *r* and global phase $\chi$), we try to integrate over the two variables *r* and $\chi$ in $\Delta (r,\chi ,\theta ,\varphi )$ . Considering the integral measure $d^{2}\alpha d^{2}\beta \equiv \frac{1}{4} r^{3}\sin \theta drd\chi d\theta d\varphi$, the integration must be carried out in two steps. In the first step, we remove the physically irrelevant global phase $\chi$by using $\delta (k-k^{\prime })=\int _{0}^{2\pi }d\chi e^{i\chi (k-k^{\prime })}$ and have

8 $$\begin{aligned}{}&\Delta (r,\theta ,\varphi ) \\= & {} \int _{0}^{2\pi }d\chi \Delta (r,\chi ,\theta ,\varphi ) \\= & {} \frac{1}{\pi ^{2}}e^{-2r^{2}}:\overset{\infty }{\underset{k=0}{ \sum }}\frac{r^{2k}[4(\cos \frac{\theta }{2}a^{\dag }+e^{-i\varphi }\sin \frac{\theta }{2}b^{\dag })(\cos \frac{\theta }{2}a+e^{i\varphi }\sin \frac{ \theta }{2}b)]^{k}}{(k!)^{2}} \\&\times \exp [-2(a^{\dag }a+b^{\dag }b)]:. \end{aligned}$$

Next, we integrate over the radial variable *r* to get

9 $$\begin{aligned}{}&\Delta (\theta ,\varphi ) \\= & {} 4\int _{0}^{\infty }drr^{3}\Delta (r,\theta ,\varphi ) \\= & {} \frac{4}{\pi ^{2}}:\int _{0}^{\infty }drr^{2k+3}e^{-2r^{2}} \\&\times \overset{\infty }{\underset{k=0}{\sum }}\frac{2^{2k}[(\cos \frac{ \theta }{2}a^{\dag }+e^{-i\varphi }\sin \frac{\theta }{2}b^{\dag })(\cos \frac{\theta }{2}a+e^{i\varphi }\sin \frac{\theta }{2}b)]^{k}}{(k!)^{2}} \\&\times \exp [-2(a^{\dag }a+b^{\dag }b)]:. \end{aligned}$$

By using the integral formula $\int _{0}^{\infty }dxx^{2k+3}e^{-ax^{2}}=\frac{ (k+1)!}{2a^{k+2}}(a>0,k>0)$, we have

10 $$\begin{aligned}{}&\Delta (\theta ,\varphi ) \\= &{} \frac{1}{2\pi ^{2}}:\overset{\infty }{\underset{k=0}{\sum }}(k+1) \frac{[(\cos \frac{\theta }{2}a^{\dag }+e^{-i\varphi }\sin \frac{\theta }{2} b^{\dag })(\cos \frac{\theta }{2}a+e^{i\varphi }\sin \frac{\theta }{2}b)]^{k} }{k!} \\&\times \exp [-2(a^{\dag }a+b^{\dag }b)]: \\= & {} \frac{1}{2\pi ^{2}}:[2(\cos \frac{\theta }{2}a^{\dag }+e^{-i\varphi }\sin \frac{\theta }{2}b^{\dag })(\cos \frac{\theta }{2} a+e^{i\varphi }\sin \frac{\theta }{2}b)+1] \\&\times \exp [2(\cos \frac{\theta }{2}a^{\dag }+e^{-i\varphi }\sin \frac{ \theta }{2}b^{\dag })(\cos \frac{\theta }{2}a+e^{i\varphi }\sin \frac{\theta }{2}b)] \\&\times \exp [-2(a^{\dag }a+b^{\dag }b)]:. \\= & {} \frac{1}{2\pi ^{2}}:[2(\cos \frac{\theta }{2}a^{\dag }+e^{-i\varphi }\sin \frac{\theta }{2}b^{\dag })(\cos \frac{\theta }{2} a+e^{i\varphi }\sin \frac{\theta }{2}b)+1] \\&\times \exp [\cos \theta (a^{\dag }a-b^{\dag }b)+\sin \theta (e^{-i\varphi }a^{\dag }b+e^{i\varphi }b^{\dag }a)-(a^{\dag }a+b^{\dag }b)]: \end{aligned}$$

Thus, we have derived polarization related Wigner operator $\Delta (\theta ,\varphi )$ via suitable marginals of distributions for the field quadratures by removing the degrees of freedom irrelevant for the specification of polarization, which has at least two significant meanings according to Ref.^8^. On the one hand, $\Delta (\theta ,\varphi )$ have an exact correspondence with polarization in classical optics. On the other hand, polarization related Wigner functions provide a feasible approach to examine and measure diverse polarization properties by using diverse experimental procedures, such as homodyne and heterodyne detection, tomography, and atom-field interactions.

When $\theta =0$, from the Eq. (10), we get

11 $$\begin{aligned} \Delta (0,\varphi )=\frac{1}{2\pi ^{2}}:(2a^{\dag }a+1)e^{-2b^{\dag }b}:=(2a^{\dag }a+1)(-1)^{b^{\dag }b}. \end{aligned}$$

Now, we introduce the operator

12 $$\begin{aligned} U(\zeta )=\exp (\zeta J_{+}-\zeta ^{*}J_{-}),\,\,\zeta =\frac{ \theta }{2}e^{-i\varphi }, \end{aligned}$$

which is defined in terms of the two mode realization of the SU(2) algebra

13 $$\begin{aligned} J_{+}=a^{\dag }b,\quad J_{-}=b^{\dag }a,\quad J_{0}=\frac{1 }{2}(a^{\dag }a-b^{\dag }b), \end{aligned}$$

with commutation relations

14 $$\begin{aligned}{}[J_{0},J_{\pm }]=\pm J_{\pm },\quad[J_{+},J_{-}]=2J_{0}. \end{aligned}$$

$U(\zeta )$ is the unitary operator representing SU(2) transformation and its normally ordered form is

15 $$\begin{aligned} U(\zeta )= & {} \int \int \frac{dz_{1}^{2}dz_{2}^{2}}{\pi ^{2}}\left| \mathbf {M }\left( {\begin{array}{c}z_{1}\\ z_{2}\end{array}}\right) \right\rangle \left\langle \left( {\begin{array}{c}z_{1}\\ z_{2}\end{array}}\right) \right| \\= & {} :\exp [(a^{\dag },b^{\dag })(\mathbf {M}-\mathbf {1})\left( {\begin{array}{c}a\\ b\end{array}}\right) ]:, \end{aligned}$$

with

16 $$\begin{aligned} \mathbf {M=}\left( \begin{array}{cc} \cos \frac{\theta }{2} &{} -e^{-i\varphi }\sin \frac{\theta }{2} \\ e^{i\varphi }\sin \frac{\theta }{2} &{} \cos \frac{\theta }{2} \end{array} \right) , \end{aligned}$$

so that

17 $$\begin{aligned} U(\zeta )\left( {\begin{array}{c}a^{\dag }\\ b^{\dag }\end{array}}\right) U^{\dag }(\zeta )=\mathbf {M}^{-1}\left( {\begin{array}{c} a^{\dag }\\ b^{\dag }\end{array}}\right) ,\mathbf {M}^{-1}\mathbf {=}\left( \begin{array}{cc} \cos \frac{\theta }{2} &{} e^{-i\varphi }\sin \frac{\theta }{2} \\ -e^{i\varphi }\sin \frac{\theta }{2} &{} \cos \frac{\theta }{2} \end{array} \right) . \end{aligned}$$

Our purpose is to prove that

18 $$\begin{aligned} \Delta (\theta ,\varphi )=U(\zeta )\Delta (0,\varphi )U^{\dag }(\zeta ). \end{aligned}$$

In fact, according to Eq. (10), we only need to show that

19 $$\begin{aligned}{}&U(\zeta )(-1)^{b^{\dag }b}U^{\dag }(\zeta ) \\= & {} \frac{1}{2\pi ^{2}}:\exp [\cos \theta (a^{\dag }a-b^{\dag }b)+\sin \theta (e^{-i\varphi }a^{\dag }b+e^{i\varphi }b^{\dag }a) \\&-(a^{\dag }a+b^{\dag }b)]:. \end{aligned}$$

The parity operator $(-1)^{b^{\dag }b}$ can be written as

20 $$\begin{aligned}{}&(-1)^{b^{\dag }b} \\= & {} \int \int \frac{dz_{1}^{2}dz_{2}^{2}}{\pi ^{2}}|z_{1},-z_{2}\rangle \langle z_{1},z_{2}| \\= & {} \int \int \frac{dz_{1}^{2}dz_{2}^{2}}{\pi ^{2}}\left| \left( \begin{array}{cc} 1 &{} 0 \\ 0 &{} -1 \end{array} \right) \left( {\begin{array}{c}z_{1}\\ z_{2}\end{array}}\right) \right\rangle \left\langle \left( {\begin{array}{c}z_{1}\\ z_{2}\end{array}}\right) \right| \\= & {} \int \int \frac{dz_{1}^{2}dz_{2}^{2}}{\pi ^{2}}\left| \mathbf {\sigma } _{z}\left( {\begin{array}{c}z_{1}\\ z_{2}\end{array}}\right) \right\rangle \left\langle \left( {\begin{array}{c}z_{1}\\ z_{2}\end{array}}\right) \right| \\= & {} :\exp [(a^{\dag },b^{\dag })(\mathbf {\sigma }_{z}-\mathbf {1})\left( {\begin{array}{c} a\\ b\end{array}}\right) ]:. \end{aligned}$$

Utilizing the bosonic operator realization of normally ordered product form of $SU_{n}$ group^11,12^, we have

21 $$\begin{aligned}{}&:\exp [(a^{\dag },b^{\dag })(\mathbf {u}^{\prime }-\mathbf {1})\left( {\begin{array}{c}a \\ b\end{array}}\right) ]::\exp [(a^{\dag },b^{\dag })(\mathbf {u}-\mathbf {1})\left( {\begin{array}{c}a \\ b\end{array}}\right) ]:\nonumber \\= & {} :\exp [(a^{\dag },b^{\dag })(\mathbf {u}^{\prime }\mathbf {u}-\mathbf { 1})\left( {\begin{array}{c}a\\ b\end{array}}\right) ]:. \end{aligned}$$

Thus, combining Eqs. (15), (20) and (21), we can get

22 $$\begin{aligned}{}&U(\zeta )(-1)^{b^{\dag }b}U^{\dag }(\zeta ) \\= & {} :\exp [(a^{\dag },b^{\dag })(\mathbf {M\sigma }_{z}\mathbf {M}^{-1}- \mathbf {1})\left( {\begin{array}{c}a\\ b\end{array}}\right) ]:\nonumber \\= & {} :\exp [(a^{\dag },b^{\dag })\left( \begin{array}{cc} \cos \theta -1 &{} e^{-i\varphi }\sin \theta \\ e^{i\varphi }\sin \theta &{} -\cos \theta -1 \end{array} \right) \left( {\begin{array}{c}a\\ b\end{array}}\right) ]:\nonumber \\= & {} :\exp [\cos \theta (a^{\dag }a-b^{\dag }b)+\sin \theta (e^{-i\varphi }a^{\dag }b+e^{i\varphi }b^{\dag }a) \\&-(a^{\dag }a+b^{\dag }b)]:. \end{aligned}$$

So, Eq. (19) is proved, which also shows that the two-mode Wigner operator that integrates over the variables *r* and $\chi$ is indeed SU(2) symmetric.

For the coherent state $|z_{1},z_{2}\rangle$, we can get its Wigner function immediately

23 $$\begin{aligned}{}&W_{|z_{1},z_{2}\rangle }(\theta ,\varphi ) \\= & {} Tr[|z_{1},z_{2}\rangle \langle z_{1},z_{2}|\Delta (\theta ,\varphi )] \\= & {} \langle z_{1},z_{2}|\Delta (\theta ,\varphi )|z_{1},z_{2}\rangle \\= & {} \frac{1}{2\pi ^{2}}\langle z_{1},z_{2}|:[2(\cos \frac{\theta }{2}a^{\dag }+e^{-i\varphi }\sin \frac{\theta }{2}b^{\dag })(\cos \frac{ \theta }{2}a+e^{i\varphi }\sin \frac{\theta }{2}b)+1] \\&\times \exp [\cos \theta (a^{\dag }a-b^{\dag }b)+\sin \theta (e^{-i\varphi }a^{\dag }b+e^{i\varphi }b^{\dag }a)-(a^{\dag }a+b^{\dag }b)]:|z_{1},z_{2}\rangle \\= & {} [2(\cos \frac{\theta }{2}z_{1}^{*}+e^{-i\varphi }\sin \frac{\theta }{2 }z_{2}^{*})(\cos \frac{\theta }{2}z_{1}+e^{i\varphi }\sin \frac{\theta }{ 2}z_{2})+1] \\&\times \exp [\cos \theta (|z_{1}|^{2}-|z_{2}|^{2})+\sin \theta (e^{-i\varphi }z_{1}^{*}z_{2}+e^{i\varphi }z_{2}^{*}z_{1}) \\&-(|z_{1}|^{2}+|z_{2}|^{2})] \end{aligned}$$

For other pure states, as long as the inner product of them and the coherent state can be given, in principle, the Wigner function can be calculated by inserting the completeness relations of the coherent states. Furthermore, for any mixed state, as long as we calculate the expected value of the pure state of its subsystem, we can immediately get its Wigner function.

## Two-mode Wigner operator for SU(1,1)

Next, we use the parametrization

24 $$\begin{aligned} \alpha =re^{i(\chi +\varphi )/2}\cosh \frac{\tau }{2},\beta =re^{i(\chi -\varphi )/2}\sinh \frac{\tau }{2}, \end{aligned}$$

where the radial variable $r^{2}=|\alpha |^{2}-|\beta |^{2}$ represents the difference in intensities between the two modes, and the parameters $\chi$ and $\tau$ can be interpreted as azimuthal and polar angles on a two-sheeted hyperboloid^10^.

Substituting Eq. (24) into Eq. (4), $\Delta (\alpha ,\beta )$ can be rewritten as

25 $$\begin{aligned}{}&\Delta (r,\chi ,\varphi ,\tau ) \\ \equiv & {} \Delta (\alpha ,\beta ) \\= & {} \frac{1}{\pi ^{2}}e^{-2\cosh \tau r^{2}}:\exp [2re^{i\varphi /2}(e^{i\chi /2}\cosh \frac{\tau }{2}a^{\dag }+e^{-i\chi /2}\sinh \frac{\tau }{2}b^{\dag }) \\&+2re^{-i\varphi /2}(e^{-i\chi /2}\cosh \frac{\tau }{2}a+e^{i\chi /2}\sinh \frac{\tau }{2}b)]\exp [-2(a^{\dag }a+b^{\dag }b)]:\nonumber \\= & {} \frac{1}{\pi ^{2}}e^{-2\cosh \tau r^{2}} \\&\times :\overset{\infty }{\underset{k,k^{\prime }=0}{\sum }}\frac{ (2r)^{k+k^{\prime }}[(e^{i\chi /2}\cosh \frac{\tau }{2}a^{\dag }+e^{-i\chi /2}\sinh \frac{\tau }{2}b^{\dag })]^{k}[(e^{-i\chi /2}\cosh \frac{\tau }{2} a+e^{i\chi /2}\sinh \frac{\tau }{2}b)]^{k^{\prime }}}{k!k^{\prime }!} \\&\times e^{i\varphi (k-k^{\prime })/2}\exp [-2(a^{\dag }a+b^{\dag }b)]:. \end{aligned}$$

We proceed to integrate over the physically irrelevant phase $\varphi$ to get

26 $$\begin{aligned}{}&\Delta (r,\chi ,\tau ) \\= & {} \frac{1}{\pi ^{2}}:\overset{\infty }{\underset{k=0}{\sum }} r^{2k}e^{-2\cosh \tau r^{2}} \\&\times \frac{2^{2k}[(e^{i\chi /2}\cosh \frac{\tau }{2}a^{\dag }+e^{-i\chi /2}\sinh \frac{\tau }{2}b^{\dag })(e^{-i\chi /2}\cosh \frac{\tau }{2} a+e^{i\chi /2}\sinh \frac{\tau }{2}b)]^{k}}{(k!)^{2}} \\&\times \exp [-2(a^{\dag }a+b^{\dag }b)]:. \end{aligned}$$

Finally, we integrate over *r*

27 $$\begin{aligned}{}&\Delta (\chi ,\tau ) \\= & {} \frac{2}{\pi ^{2}}:\overset{\infty }{\underset{k=0}{\sum }} \int _{0}^{\infty }drr^{2k+1}e^{-2\cosh \tau r^{2}} \\&\times \frac{2^{2k}[(e^{i\chi /2}\cosh \frac{\tau }{2}a^{\dag }+e^{-i\chi /2}\sinh \frac{\tau }{2}b^{\dag })(e^{-i\chi /2}\cosh \frac{\tau }{2} a+e^{i\chi /2}\sinh \frac{\tau }{2}b)]^{k}}{(k!)^{2}} \\&\times \exp [-2(a^{\dag }a+b^{\dag }b)]:\nonumber \\= & {} \frac{1}{2\pi ^{2}\cosh \tau }:\exp [\frac{2}{\cosh \tau }(e^{i\chi /2}\cosh \frac{\tau }{2}a^{\dag }+e^{-i\chi /2}\sinh \frac{\tau }{2}b^{\dag }) \\&\times (e^{-i\chi /2}\cosh \frac{\tau }{2}a+e^{i\chi /2}\sinh \frac{\tau }{ 2}b)-2(a^{\dag }a+b^{\dag }b)]:\nonumber \\= & {} \frac{1}{2\pi ^{2}\cosh \tau }:\exp [\sec h\theta (a^{\dag }a-b^{\dag }b)+\tanh \theta (e^{i\chi }a^{\dag }b^{\dag }+e^{-i\chi }ab) \\&-(a^{\dag }a+b^{\dag }b)]: \end{aligned}$$

Now, we introduce the two-mode squeezed operator

28 $$\begin{aligned} S(\eta )=\exp (\eta K_{+}-\eta ^{*}K_{-}),\quad \eta =\frac{\tau }{2}e^{i\chi }, \end{aligned}$$

which is defined in terms of the two mode realization of the SU(1,1) algebra

29 $$\begin{aligned} K_{+}=a^{\dag }b^{\dag }, \quad K_{-}=ab,\quad K_{0}=\frac{1 }{2}(a^{\dag }a+b^{\dag }b+1), \end{aligned}$$

with commutation relations

30 $$\begin{aligned}{}[K_{0},K_{\pm }]=\pm K_{\pm },\quad[K_{-},K_{+}]=2K_{0}. \end{aligned}$$

Now, we try to show that the two-mode Wigner operator $\Delta (\chi ,\tau )$ has SU(1,1) symmetric form, that is

31 $$\begin{aligned} \Delta (\chi ,\tau )=S(\eta )(-1)^{b^{\dag }b}S^{\dag }(\eta ). \end{aligned}$$

Here, it should be pointed out that the form given by Eq. (24) in the Ref.^10^ is wrong. The SU(1,1) parity $(-1)^{K_{0}}$ there should be replaced by the single mode parity operator $(-1)^{b^{\dag }b}$ here.

According to the Baker-Campell-Hausdorff (BCH) formula

32 $$\begin{aligned} S(\eta )b^{\dag }S^{\dag }(\eta )= & {} b^{\dag }\cosh \frac{\tau }{2} -ae^{-i\chi }\sinh \frac{\tau }{2}, \\ S(\eta )bS^{\dag }(\eta )= & {} b\cosh \frac{\tau }{2}-a^{\dag }e^{i\chi }\sinh \frac{\tau }{2}, \end{aligned}$$

which is a similar transformation. The parity operator $(-1)^{b^{\dag }b}$ is written in the Weyl ordering^13^ as

33 $$\begin{aligned} (-1)^{b^{\dag }b}= \begin{array}{c} :\\ :\end{array} \delta (b^{\dag })\delta (b) \begin{array}{c} :\\ :\end{array} , \end{aligned}$$

where $$\begin{array}{c} :\\ :\end{array} \begin{array}{c} :\\ :\end{array}$$ represents the Weyl ordering. Since in Ref.^14,15^, the authors have proved that a similar transformation *S* does not disturb the Weyl ordering or we can say that it keeps the Weyl ordering invariant, this means that

34 $$\begin{aligned} S \begin{array}{c} :\\ :\end{array} (\cdots ) \begin{array}{c} :\\ :\end{array} S^{-1}= \begin{array}{c} :\\ :\end{array} S(\cdots )S^{-1} \begin{array}{c} :\\ :\end{array} . \end{aligned}$$

Because of this property, we have

35 $$\begin{aligned}{}&S(\eta )(-1)^{b^{\dag }b}S^{\dag }(\eta ) \\= & {} S(\eta ) \begin{array}{c} :\\ :\end{array} \delta (b^{\dag })\delta (b) \begin{array}{c} :\\ :\end{array} S^{\dag }(\eta ) \\= & {} \begin{array}{c} :\\ :\end{array} \delta (b^{\dag }\cosh \frac{\tau }{2}-ae^{-i\chi }\sinh \frac{\tau }{2} )\delta (b\cosh \frac{\tau }{2}-a^{\dag }e^{i\chi }\sinh \frac{\tau }{2}) \begin{array}{c} :\\ :\end{array} . \end{aligned}$$

By using the formula as follows^14,15^

36 $$\begin{aligned}{}&\begin{array}{c} :\\ :\end{array} g(a,a^{\dag }) \begin{array}{c} :\\ :\end{array} \\= & {} \int _{-\infty }^{\infty }d^{2}\alpha g(\alpha ,\alpha ^{*})\Delta (\alpha ,\alpha ^{*}) \\= & {} \frac{1}{\pi }\int _{-\infty }^{\infty }d^{2}\alpha g(\alpha ,\alpha ^{*}):e^{-2(\alpha ^{*}-a^{\dag })(\alpha -a)}:, \end{aligned}$$

Eq. (35) can be transformed into

37 $$\begin{aligned}{}&S(\eta )(-1)^{b^{\dag }b}S^{\dag }(\eta ) \\= & {} \frac{1}{\pi ^{2}}\int \int d^{2}\alpha d^{2}\beta \delta \left(\beta ^{*}\cosh \frac{\tau }{2}-\alpha e^{-i\chi }\sinh \frac{\tau }{2}\right)\delta \left(\beta \cosh \frac{\tau }{2}-\alpha ^{*}e^{i\chi }\sinh \frac{\tau }{2}\right) \\&\times :e^{-2(\alpha ^{*}-a^{\dag })(\alpha -a)}e^{-2(\beta ^{*}-b^{\dag })(\beta -b)}:\nonumber \\= & {} \frac{1}{\pi ^{2}\cosh ^{2}\frac{\tau }{2}}:\int d^{2}\alpha \exp \left[-2(\alpha ^{*}-a^{\dag })(\alpha -a)-2\left(\alpha e^{-i\chi }\tanh \frac{ \tau }{2}-b^{\dag }\right)\left(\alpha ^{*}e^{i\chi }\tanh \frac{\tau }{2}-b\right)\right]:\nonumber \\= & {} \frac{1}{\pi ^{2}\cosh ^{2}\frac{\tau }{2}}:\int d^{2}\alpha \exp \left[-2|\alpha |^{2}\left(1+\tanh ^{2}\frac{\tau }{2}\right) \right.\nonumber \\&\left.+2\alpha \left(a^{\dag }+e^{-i\chi }\tanh \frac{\tau }{2}b\right)+2\alpha ^{*}\left(a+e^{i\chi }\tanh \frac{\tau }{2}b^{\dag }\right)-2(a^{\dag }a+b^{\dag }b)\right]:\nonumber \\= &{} \frac{1}{2\pi ^{2}\cosh \tau }:\exp \left[\frac{2(a^{\dag }+e^{-i\chi }\tanh \frac{\tau }{2}b)(a+e^{i\chi }\tanh \frac{\tau }{2}b^{\dag })}{ 1+\tanh ^{2}\frac{\tau }{2}}-2(a^{\dag }a+b^{\dag }b)\right]:\nonumber \\= &{} \frac{1}{2\pi ^{2}\cosh \tau }:\exp [\sec h\theta (a^{\dag }a-b^{\dag }b)+\tanh \theta (e^{i\chi }a^{\dag }b^{\dag }+e^{-i\chi }ab)-(a^{\dag }a+b^{\dag }b)]:, \end{aligned}$$

where we used the integral formula $\int \frac{d^{2}\alpha }{\pi ^{2}}\exp [-h|\alpha |^{2}+s\alpha +\eta \alpha ^{*}]=\frac{1}{h}\exp (\frac{s\eta }{h}),({\hbox {Re}}\,h<0)$. Eq. (37) is exactly the same as Eq. (27), so Eq. (31) is proved.

## Two-mode Wigner operator in polar coordinates

### Two-dimensional isotropic harmonic oscillator

The Hamiltonian of a two-dimensional isotropic harmonic oscillator system can be written as

38 $$\begin{aligned} H=\frac{1}{2}(X^{2}+P_{x}^{2}+Y^{2}+P_{y}^{2}). \end{aligned}$$

The corresponding creation and annihilation operators are

39 $$\begin{aligned}{}&a^{\dag }=\frac{1}{\sqrt{2}}(X-iP_{x}),\,\,a=\frac{1}{\sqrt{2}} (X+iP_{x}), \end{aligned}$$

40 $$\begin{aligned}{}&\quad b^{\dag }=\frac{1}{\sqrt{2}}(Y-iP_{y}),\,\,a=\frac{1}{\sqrt{2}} (Y+iP_{y}) \end{aligned}$$

and the total number operator

41 $$\begin{aligned} N=N_{1}+N_{2}. \end{aligned}$$

Then, the eigenstate of the Hamiltonian $H=N+1$ is

42 $$\begin{aligned} |n_{x},n_{y}\rangle =(n_{x}!n_{y}!)^{-\frac{1}{2}}a^{\dag n_{x}}b^{\dag n_{y}}|0,0\rangle . \end{aligned}$$

Choosing a Cartesian coordinate frame is not the only way to describe the problem. Since the energy in the rotating plane $x-y$ is conserved, we can also choose other rotating reference frames. The angular momentum operator *L* is defined as

43 $$\begin{aligned} L=XP_{y}-YP_{x}=i(ab^{\dag }-a^{\dag }b). \end{aligned}$$

It should be noted that $\{N,L\}$ are a set of commutated observables. Setting

44 $$\begin{aligned}{}&a_{\pm }=\frac{1}{\sqrt{2}}(a\mp ib), \end{aligned}$$

45 $$\begin{aligned}{}&a_{\pm }^{\dag }=\frac{1}{\sqrt{2}}(a^{\dag }\pm ib^{\dag }), \end{aligned}$$

we also treat $a_{\pm }$ and $a_{\pm }^{\dag }$ as creation and annihilation operators, whose corresponding number operators are $N_{\pm }=a_{\pm }^{\dag }a_{\pm }$. The two commutated observables $N_{+}$ and $N_{-}$ form a set of complete basis. For each group of eigenvalues $(n_{+},n_{-})$, there exists an eigenvector, which is marked with $|n_{+},n_{-}\rangle$ and

46 $$\begin{aligned} |n_{+},n_{-}\rangle =(n_{+}!n_{-}!)^{-\frac{1}{2}}a_{+}^{\dag n_{+}}a_{-}^{\dag n_{-}}|0,0\rangle , \end{aligned}$$

which constitutes a complete set of orthogonal eigenvectors. One can find

47 $$\begin{aligned} N=N_{+}+N_{-} \end{aligned}$$

and

48 $$\begin{aligned} L=N_{+}-N_{-}, \end{aligned}$$

which shows that the two commutated observables *N* and *L* form a set of complete basis.

### Two-dimensional coordinate eigenstates in polar coordinates

We know that in the Cartesian coordinates, the two-dimensional coordinate eigenstate is descriped as

49 $$\begin{aligned} |x,y\rangle =\pi ^{-\frac{1}{2}}\exp \left[-\frac{1}{2}(x^{2}+y^{2})+\sqrt{2} (xa^{\dag }+yb^{\dag })-\frac{a^{\dag 2}+b^{\dag 2}}{2}\right]|0,0\rangle . \end{aligned}$$

Now we write the Eq. (49) as a form in polar coordinates^16^. Setting

50 $$\begin{aligned} x= & {} r\cos \phi , \\ y= & {} r\sin \phi , \\ x\pm iy= & {} re^{\pm i\phi }. \end{aligned}$$

Substituting Eqs. (44), (45) and (50) into Eq. (49), we have

51 $$\begin{aligned}{}&|r,\phi \rangle \\ \equiv & {} |x,y\rangle \\= & {} \pi ^{-\frac{1}{2}}\exp \left\{-\frac{1}{2}r^{2}+\sqrt{2}r\cos \phi \left(\frac{ a_{+}^{\dag }+a_{-}^{\dag }}{\sqrt{2}}\right)+\sqrt{2}r\sin \phi \left(\frac{ a_{+}^{\dag }-a_{-}^{\dag }}{\sqrt{2}i}\right) \right.\nonumber \\&\left.-\frac{1}{2}\left[\left(\frac{a_{+}^{\dag }+a_{-}^{\dag }}{\sqrt{2}}\right)^{2}+\left(\frac{ a_{+}^{\dag }-a_{-}^{\dag }}{\sqrt{2}i}\right)^{2}\right]\right\}|0,0\rangle \\= & {} \pi ^{-\frac{1}{2}}\exp \left(-\frac{1}{2}r^{2}+re^{-i\phi }a_{+}^{\dag }+re^{i\phi }a_{-}^{\dag }-a_{+}^{\dag }a_{-}^{\dag }\right)|0,0\rangle \\ \equiv & {} |\xi \rangle ,\,\,\xi =re^{-i\phi }. \end{aligned}$$

Eq. (51) is a very important result, because $|\xi \rangle$ is actually the entangled state representation created by Fan^17,18^. $|\xi \rangle$ is the continuous variable version of the EPR entangled state, which is introduced in the following way.

Since $[X_{+}+X_{-},P_{+}-P_{-}]=0$, where $X_{\pm }$ and $P_{\pm }$ are the position and momentum operators of the rotating reference frame, respectively, we can give the common eigenstate $|\xi \rangle$ of $X_{+}+X_{-}$ and $P_{+}-P_{-}$, that is

52 $$\begin{aligned} (X_{+}+X_{-})|\xi \rangle= & {} \sqrt{2}{\hbox {Re}}\,(\xi )|\xi \rangle , \\ (P_{+}-P_{-})|\xi \rangle= & {} \sqrt{2}{\hbox {Im}}\,(\xi )|\xi \rangle . \end{aligned}$$

By using the method of IWOP, we can prove its orthogonality and completeness

53 $$\begin{aligned} \langle \xi ^{\prime }|\xi \rangle =\pi \delta ^{(2)}(\xi ^{\prime }-\xi ) \end{aligned}$$

and

54 $$\begin{aligned} \frac{1}{\pi }\int d^{2}\xi |\xi \rangle \langle \xi |=1. \end{aligned}$$

In Ref.^16^, we have shown that

55 $$\begin{aligned} \langle r,\phi |n_{+},n_{-}\rangle =\sqrt{\frac{n_{-}!}{n_{+}!}}e^{-\frac{ r^{2}}{2}}r^{l}L_{\frac{n-l}{2}}^{l}(r^{2})e^{il\phi }, \end{aligned}$$

where we have set

56 $$\begin{aligned} N|n_{+},n_{-}\rangle= & {} (n_{+}+n_{-})|n_{+},n_{-}\rangle =n|n_{+},n_{-}\rangle , \\ L|n_{+},n_{-}\rangle= & {} (n_{+}-n_{-})|n_{+},n_{-}\rangle =l|n_{+},n_{-}\rangle . \end{aligned}$$

The RHS of Eq. (55) is a standard Laguerre–Gaussian mode in quantum optics.

### Two-dimensional momentum eigenstates in polar coordinates

Let’s explore another entangled state representation^17,18^

57 $$\begin{aligned} |\eta \rangle =\exp (-\frac{1}{2}|\eta |^{2}+\eta a_{+}^{\dag }-\eta ^{*}a_{-}^{\dag }+a_{+}^{\dag }a_{-}^{\dag })|0,0\rangle ,\,\,\eta =pe^{i\theta }, \end{aligned}$$

Then, one can immediately check the following relations

58 $$\begin{aligned} (X_{+}-X_{-})|\eta \rangle= & {} \sqrt{2}{\hbox {Re}}\,(\eta )|\eta \rangle , \\ (P_{+}+P_{-})|\eta \rangle= & {} \sqrt{2}{\hbox {Im}}\,(\eta )|\eta \rangle . \end{aligned}$$

$|\eta \rangle$ is the common eigenstate of $X_{+}-X_{-}$ and $P_{+}+P_{-}$ , where $X_{\pm }$ and $P_{\pm }$ are the quadrature operators. We can also find

59 $$\begin{aligned} (a_{+}-a_{-}^{\dag })|\eta \rangle= & {} \eta |\eta \rangle , \\ (a_{+}^{\dag }-a_{-})|\eta \rangle= & {} \eta ^{*}|\eta \rangle . \end{aligned}$$

Then, we have

60 $$\begin{aligned} \sqrt{\frac{a_{+}-a_{-}^{\dag }}{a_{+}^{\dag }-a_{-}}}|\eta \rangle =\sqrt{ \frac{\eta }{\eta ^{*}}}|\eta \rangle =e^{i\theta }|\eta \rangle . \end{aligned}$$

According to Eqs. (39), (40), (44) and (45), we get

61 $$\begin{aligned}{}&\sqrt{\frac{a_{+}-a_{-}^{\dag }}{a_{+}^{\dag }-a_{-}}} \\= & {} \sqrt{\frac{(a-a^{\dag })+i(b^{\dag }-b)}{(a^{\dag }-a)+i(b^{\dag }-b)}} \\= & {} \frac{P_{y}+iP_{x}}{\sqrt{P_{x}^{2}+P_{y}^{2}}} \end{aligned}$$

and

62 $$\begin{aligned}{}&\frac{P_{y}+iP_{x}}{\sqrt{P_{x}^{2}+P_{y}^{2}}}|p_{x},p_{y}\rangle \\= & {} \frac{p_{y}+ip_{x}}{\sqrt{p_{x}^{2}+p_{y}^{2}}}|p_{x},p_{y}\rangle \\= & {} e^{i\theta }|\eta \rangle ,\,\,\,\theta =\arctan \frac{p_{y}}{p_{x}}. \end{aligned}$$

From Eqs. (61) and (62), We can conjecture $|\eta \rangle \equiv |p_{x},p_{y}\rangle$, where $|p_{x},p_{y}\rangle$ is two-dimensional momentum eigenstate. Now we set $p=\sqrt{p_{x}^{2}+p_{y}^{2}}\equiv |\eta |$ and have

63 $$\begin{aligned}{}&|p_{x},p_{y}\rangle \\= & {} \pi ^{-\frac{1}{2}}\exp \left[-\frac{1}{2}(p_{x}^{2}+p_{y}^{2})+\sqrt{2} i(p_{x}a^{\dag }+p_{y}b^{\dag })+\frac{a^{\dag 2}+b^{\dag 2}}{2}\right]|0,0\rangle \\= & {} \pi ^{-\frac{1}{2}}\exp \left[-\frac{1}{2}p^{2}+\sqrt{2}ip\sin \theta \left(\frac{ a_{+}^{\dag }+a_{-}^{\dag }}{\sqrt{2}}\right)+\sqrt{2}ip\cos \theta \left(\frac{ a_{+}^{\dag }-a_{-}^{\dag }}{\sqrt{2}i}\right)\right. \\&\left.+a_{+}^{\dag }a_{-}^{\dag }\right]|0,0\rangle \\= & {} \pi ^{-\frac{1}{2}}\exp \left(-\frac{1}{2}p^{2}+pe^{i\theta }a_{+}^{\dag }-pe^{-i\theta }a_{-}^{\dag }+a_{+}^{\dag }a_{-}^{\dag }\right)|0,0\rangle . \end{aligned}$$

The result given by Eq. (63) shows that the entangled state representation $|\eta \rangle$ is exactly the form of the two-dimensional momentum eigenstate $|p_{x},p_{y}\rangle$ in polar coordinates.

### Two-mode Wigner operator in polar coordinates

In Ref.^16^, we have demonstrated that two-mode Wigner operator

64 $$\begin{aligned} \Delta (\alpha ,\beta )=\Delta (\alpha _{+},\alpha _{-}), \end{aligned}$$

which is a meaningful result. However, as mentioned above, two-mode Wigner operator can be converted into multiple forms by setting parameters with different physical meanings. In this section, we still start from the normally ordered form of two-mode Wigner operator to discuss a more meaningful result.

Substituting $\alpha =\frac{x+ip_{x}}{\sqrt{2}}$ and $\beta =\frac{y+ip_{y}}{ \sqrt{2}}$ into Eq. (4), we can recast the Wigner operator $\Delta (\alpha ,\beta )$ as the following version

65 $$\begin{aligned}{}&\Delta (r,\phi ,p,\theta ) \\ \equiv & {} \Delta (\alpha ,\beta ) \\= & {} \frac{1}{\pi ^{2}}:\exp \{-(r^{2}+p^{2})+\sqrt{2}r[(a^{\dag }+a)\cos \phi +(b^{\dag }+b)\sin \phi ] \\&+\sqrt{2}ip[(a^{\dag }-a)\sin \theta +(b^{\dag }-b)\cos \theta ]-2(a^{\dag }a+b^{\dag }b)\}:. \end{aligned}$$

Now we try to integrate over *p* and $\theta$. Noticing that the integral measure $d^{2}\eta \sim 2pdpd\theta$, we obtain

66 $$\begin{aligned}{}&\Delta (r,\phi ) \\= & {} \int d^{2}\eta \Delta (r,\phi ,p,\theta ) \\= & {} \frac{1}{\pi ^{2}}:\exp \{-r^{2}+\sqrt{2}r[(a^{\dag }+a)\cos \phi +(b^{\dag }+b)\sin \phi ]-2(a^{\dag }a+b^{\dag }b)\} \\&\times \int _{0}^{\infty }2pdp\int _{0}^{2\pi }d\theta \exp \{-p^{2}+\sqrt{2} ip[(a^{\dag }-a)\sin \theta +(b^{\dag }-b)\cos \theta ]\}:\nonumber \\= & {} \frac{1}{\pi ^{2}}:\exp \{-r^{2}+\sqrt{2}r[(a^{\dag }+a)\cos \phi +(b^{\dag }+b)\sin \phi ]-2(a^{\dag }a+b^{\dag }b)\} \\&\times \int _{0}^{\infty }2pe^{-p^{2}}dp\overset{\infty }{\underset{ m=-\infty }{\sum }}J_{m}[\sqrt{2}ip(a^{\dag }-a)]\overset{\infty }{\underset{m^{\prime }=-\infty }{\sum }}J_{m^{\prime }}[\sqrt{2}ip(b^{\dag }-b)]e^{im^{\prime }\pi /2} \\&\times \int _{0}^{2\pi }e^{i(m-m^{\prime })\theta }d\theta :. \end{aligned}$$

In the derivation of Eq. (66), we have used the formula $e^{ix\sin t}= \overset{\infty }{\underset{m=-\infty }{\sum }}J_{m}(x)e^{imt}$, where $J_{m}(x)$ is Bessel function. By using $\delta (m-m^{\prime })=\int _{0}^{2\pi }e^{i(m-m^{\prime })\theta }d\theta$, we transform Eq. (66) into

67 $$\begin{aligned}{}&\Delta (r,\phi ) \\= & {} \frac{1}{\pi ^{2}}:\exp \{-r^{2}+\sqrt{2}r[(a^{\dag }+a)\cos \phi +(b^{\dag }+b)\sin \phi ]-2(a^{\dag }a+b^{\dag }b)\} \\&\times \overset{\infty }{\underset{m=-\infty }{\sum }}\int _{0}^{\infty }pe^{-p^{2}}J_{m}[\sqrt{2}ip(a^{\dag }-a)]J_{m}[\sqrt{2}ip(b^{\dag }-b)]dpe^{im\pi /2}:. \end{aligned}$$

By employing the integral formula

68 $$\begin{aligned}{}&\int _{0}^{\infty }te^{-p^{2}t^{2}}J_{\nu }(at)J_{\nu }(bt)dt \\= & {} \frac{1}{2p^{2}}\exp (-\frac{a^{2}+b^{2}}{4p^{2}})I_{\nu }(\frac{ab}{ 2p^{2}}),{\hbox {Re}}\,\nu>-1,{\hbox {Re}}\,(p^{2})>0, \end{aligned}$$

where $I_{\nu }(\alpha )=i^{-\nu }J_{\nu }(i\alpha )$, we have

69 $$\begin{aligned}{}&\Delta (r,\phi ) \\= & {} \frac{1}{\pi ^{2}}:\exp \{-r^{2}+\sqrt{2}r[(a^{\dag }+a)\cos \phi +(b^{\dag }+b)\sin \phi ]-2(a^{\dag }a+b^{\dag }b)\} \\&\times \overset{\infty }{\underset{m=-\infty }{\sum }}\frac{1}{2}\exp \left[- \frac{(a^{\dag }-a)^{2}+(b^{\dag }-b)^{2}}{2}\right]i^{-m}J_{m}\left[-\frac{i(a^{\dag }-a)(b^{\dag }-b)}{2}\right]i^{m}:\nonumber \\= & {} \frac{1}{\pi ^{2}}:\exp \{-r^{2}+\sqrt{2}r[(a^{\dag }+a)\cos \phi +(b^{\dag }+b)\sin \phi ] \\&-\frac{a^{\dag 2}+a^{2}+b^{\dag 2}+b^{2}}{2}-(a^{\dag }a+b^{\dag }b)\}:. \\= & {} \frac{1}{\pi ^{2}}\exp \left(-\frac{r^{2}}{2}+\sqrt{2}r\cos \phi a^{\dag }+ \sqrt{2}r\sin \phi b^{\dag }-\frac{a^{\dag 2}+b^{\dag 2}}{2}\right)|0,0\rangle \\&\times \langle 0,0|\exp \left(-\frac{r^{2}}{2}+\sqrt{2}r\cos \phi a+\sqrt{2} r\sin \phi b-\frac{a^{2}+b^{2}}{2}\right), \end{aligned}$$

where we have used the property of normal ordering $|0,0\rangle \langle 0,0|=:\exp [-(a^{\dag }a+b^{\dag }b)]:$. Comparing Eq. (69) with Eqs. (49) and (51), we get

70 $$\begin{aligned} \Delta (r,\phi )=|\xi \rangle \langle \xi |=\int d^{2}\eta \Delta (\alpha ,\beta ). \end{aligned}$$

By using a similar derivation to Eq. (69), we can obtain

71 $$\begin{aligned} \Delta (p,\theta )=|\eta \rangle \langle \eta |=\int d^{2}\xi \Delta (\alpha ,\beta ). \end{aligned}$$

Eqs. (70) and (71) demonstrate that the marginal distribution of the two mode Wigner operator is exactly the pure state density matrix of the entangled state representation. This conclusion first appeared in the Ref.^19^, which tells us that the marginal distributions of the Wigner function for entangled system should be understood in the sense of entanglement.

In Ref.^20^, the authors first separated $\phi$ from the Wigner operator, and then tried to use the integration method to integrate over the radial momentum $p_{r}$ in the Wigner operator. They wanted to use the above method to obtain the operator kernel function with the radius *r* and the orbital angular momentum *l* as variables, that is, $\Delta (r,p_{r},\phi ,l)\rightarrow \Delta (r,l)$, but their efforts were not successful. We notice that

72 $$\begin{aligned} p_{r}=\frac{xp_{x}+yp_{y}}{r}=p\sin (\phi +\theta ) \end{aligned}$$

and

73 $$\begin{aligned} l=xp_{y}-yp_{x}=pr\cos (\phi +\theta ). \end{aligned}$$

Since $p_{r}$ and *l* are not two independent variables, their scheme must not work.

## Concluding remarks

Using the IWOP method, we give three approaches to analyze the normal product form of the two-mode Wigner operator, which are SU(2) symmetric representation, SU(1,1) symmetric representation, and polar coordinate form. The two-mode Wigner operator has a variety of intrinsic degrees of freedom, and the IWOP method is more conducive to explaining the intrinsic relationship of these potential degrees of freedom. Our next work may be to use this approach to study the phase or angular momentum properties of the two-mode quantum state in phase space.

## Data Availability

The data that support the findings of this study are available from the corresponding author upon reasonable request.
